# Supplementary material for: History and Toxinology of Palytoxins
Source: Toxins (Basel). 2024 Sep 26;16(10):417. doi: 10.3390/toxins16100417 (PMC11511052; doi:10.3390/toxins16100417)
Supplement: Supplementary file 1 [file toxins-16-00417-s001.zip › toxins-3163534-supplementary.pdf]

toxins

MDPI

Table S1. Acute Toxicity of Palytoxins.

Organism	Sex	Strain	Source of Palytoxin *Taxonomic name* (analogue)	Route of Administration	LD_{50} (ng/kg) *unless noted	Reference
Mouse	n.s.	n.s.	*Palythoa toxica*[*]	Intraperitoneal	400	Moore et al. (1971)[1]
Mouse	n.s.	n.s.	*P. toxica*[*]	Intravenous	150	Moore et al. (1971)[1]
Mouse	n.s.	n.s.	*P. tuberculosa*[**]	Intraperitoneal	600	Kimura et al. (1973)[2]
Mouse	M	Swiss-Webster	*P. mammillosa*[***]	Intraperitoneal	50 – 100	Kaul et al. (1974)[3]
Mouse	n.s.	n.s.	*P. tuberculosa*[**]	Intravenous	530	Deguchi et al. (1974)[4]
Cat	n.s.	n.s.	*P. tuberculosa*[**]	Intravenous	200	Deguchi et al. (1974)[4]
Rabbit	M + F	New Zealand white	*P. vestitus*[†]	Intravenous	25	Wiles et al. (1974)[5]
Dog	M + F	Mongrel	*P. vestitus*[†]	Intravenous	33	Wiles et al. (1974)[5]
Monkey	M + F	Rhesus	*P. vestitus*[†]	Intravenous	78	Wiles et al. (1974)[5]
Rat	M	Wistar-Sprague Valley	*P. vestitus*[†]	Intravenous	89	Wiles et al. (1974)[5]
Guinea pig	M + F	Mongrel	*P. vestitus*[†]	Intravenous	110	Wiles et al. (1974)[5]
Mouse	M	ICR	*P. vestitus*[†]	Intravenous	450	Wiles et al. (1974)[5]
Rat	M	Wistar-Sprague Valley	*P. vestitus*[†]	Intramuscular	240	Wiles et al. (1974)[5]
Rat	M	Wistar-Sprague Valley	*P. vestitus*[†]	Subcutaneous	400	Wiles et al. (1974)[5]
Rat	M	Wistar-Sprague Valley	*P. vestitus*[†]	Intratracheal	360	Wiles et al. (1974)[5]
Mouse	F	Swiss CD-1	*P. tuberculosa*[**]	Intraperitoneal	500	Levine et al. (1987)[6]
Mouse	M	n.s.	*Radianthus macrodactylus*[‡]	Intravenous	740	Mahnir et al. (1992)[7]
Mouse	M	ddY	*Herklotsichthys quadrimaculatus*[§]	Intraperitoneal	450	Onuma et al. (1999)[8]
Mouse	n.s.	Swiss	n.s.	Intraperitoneal	720	Munday et al. (2006)[9]
Mouse	M	Swiss	*P. tuberculosa*[**] (standard)	Intraperitoneal	295	Riobo et al. (2008)[10]
Mouse	F	Swiss	*P. tuberculosa*[**] (standard)	Intraperitoneal	680	Boente-Juncal et al. (2020)[11]

Mouse	n.s.	n.s.	*Ostreopsis siamensis* (ostreocin D)	Intraperitoneal	750	Usami et al. (1995)[12]
Mouse	F	Swiss	*O. siamensis*	Intraperitoneal	720	Rhodes et al. (2002)[13]
Rat	F	Fischer F344	*P. tuberculosa*** (standard)	Intraperitoneal	1.81 µg/kg	Poli et al. (2018)[14]
Rat	F	Fischer F344	*P. toxica** (42-OH)	Intraperitoneal	1.93 µg/kg	Poli et al. (2018)[14]
Rat	F	Fischer F344	50:50 (standard: 42-OH)	Intraperitoneal	920	Poli et al. (2018)[14]
Rat	F	Fischer F344	*O. ovata* (ovatoxin-a)	Intraperitoneal	3.26 µg/kg	Poli et al. (2018)[14]
Mouse	M	ICR	*P. tuberculosa*** (standard)	Intratracheal	2 µg/kg	Ito et al. (2009)[15]
Mouse	M	ICR	*O. siamensis* (ostreocin D)	Intratracheal	11 – 13 µg/kg	Ito et al. (2009)[15]
Rat	M	Wistar	*P. tuberculosa*** (standard)	Intratracheal	5 – 7 µg/kg	Ito et al. (2009)[15]
Rat	M	Wistar	*O. siamensis* (ostreocin D)	Intratracheal	11 µg/kg	Ito et al. (2009)[15]
Rat	F	Fischer F344	*P. tuberculosa*** (standard)	Inhalational	41	Poli et al. (2018)[14]
Rat	F	Fischer F344	*P. toxica** (42-OH)	Inhalational	45	Poli et al. (2018)[14]
Rat	F	Fischer F344	50:50 (standard: 42-OH)	Inhalational	63	Poli et al. (2018)[14]
Rat	F	Fischer F344	*O. ovata* (ovatoxin-a)	Inhalational	31	Poli et al. (2018)[14]
Rat	M	Wistar-Sprague Valley	*P. vestitus*†	Intragastric	> 40	Wiles et al. (1974)[5]
Mouse	F	Swiss CD-1	*P. tuberculosa***	Gavage	767 µg/kg	Sosa et al. (2009)[16]
Mouse	F	Swiss	*P. tuberculosa*** (standard)	Gavage	599 µg/kg	Munday et al. (2006)[9]
Mouse	F	Swiss	*P. tuberculosa*** (standard)	Food Intake	> 2500 µg/kg	Munday et al. (2006)[9]

n.s = not specified; *limu-make-o-Hana; **rubbery zoanthid; ***knobby zoanthid; †button polyps; ‡sebae anemone; §goldspot herring

1. Moore, R.E. & Scheuer, P.J. Palytoxin: A New Marine Toxin from a Coelenterate. *Science* **172**, 495 - 498 (1971).
2. Kimura, S. & Hashimoto, Y. Purification of the toxin in a zoanthid *Palythoa tuberculosa*. *Publications of the Seto Marine Biological Laboratory* **20**, 713-718 (1973).
3. Kaul, P.N., Farmer, M.R. & Ciereszko, L.S. Pharmacology of palytoxin: The most potent marine toxin known. *Proceedings of the Western Pharmacology Society* **Vol. 17**, 294-301 (1974).
4. Deguchi, T., Aoshima, S. & Sakai, Y. Pharmacological actions of palythoatoxin isolated from the zoanthid, Palythoa tuberculosa. *The Japanese Journal of Pharmacology* **24**, s:116 (1974).
5. Wiles, J.S., Vick, J.A. & Christensen, M.K. Toxicological evaluation of palytoxin in several animal species. *Toxicon* **12**, 427-433 (1974).

6. Levine, L., Fujiki, H., Gjika, H.B. & Van Vunakis, H. Production of antibodies to palytoxin: neutralization of several biological properties of palytoxin. *Toxicon* **25**, 1273-1282 (1987).
7. Mahnir, V.M., Kozlovskaya, E.P. & Kalinovsky, A.I. Sea anemone Radianthus macrodactylus--a new source of palytoxin. *Toxicon* **30**, 1449-1456 (1992).
8. Onuma, Y.*, et al.* Identification of putative palytoxin as the cause of clupeotoxism. *Toxicon* **37**, 55-65 (1999).
9. Munday, R. Toxicological requirements for risk assessment of shellfish contaminants: a review. *African Journal of Marine Science* **28**, 447 - 449 (2006).
10. Riobó, P.*, et al.* Mouse bioassay for palytoxin. Specific symptoms and dose-response against dose-death time relationships. *Food Chem Toxicol* **46**, 2639-2647 (2008).
11. Boente-Juncal, A.*, et al.* Reevaluation of the acute toxicity of palytoxin in mice: Determination of lethal dose 50 (LD50) and No-observed-adverse-effect level (NOAEL). *Toxicon* **177**, 16-24 (2020).
12. Usami, M.*, et al.* Palytoxin analogs from the dinoflagellate Ostreopsis siamensis. *Journal of the American Chemical Society* **117**, 5389-5390 (1995).
13. Rhodes, L., Towers, N., Briggs, L., Munday, R. & Adamson, J. Uptake of palytoxin-like compounds by shellfish fed Ostreopsis siamensis (Dinophyceae). *New Zealand Journal of Marine and Freshwater Research* **36**, 631-636 (2002).
14. Poli, M.*, et al.* Toxicity and pathophysiology of palytoxin congeners after intraperitoneal and aerosol administration in rats. *Toxicon* **150**, 235-250 (2018).
15. Ito, E. & Yasumoto, T. Toxicological studies on palytoxin and ostreocin-D administered to mice by three different routes. *Toxicon* **54**, 244-251 (2009).
16. Sosa, S.*, et al.* Palytoxin toxicity after acute oral administration in mice. *Toxicol Lett* **191**, 253-259 (2009).
